# Non-communicable diseases and its risk factors among the transgender population in Kerala: a cross-sectional study

**DOI:** 10.1186/s12939-024-02167-7

**Published:** 2024-05-24

**Authors:** Bhavya Fernandez, Rakhal Gaitonde

**Affiliations:** Achutha Menon Centre for Health Science Studies, Sree Chitra Tirunal Institute for Medical Sciences and Technology, Trivandrum, Kerala India

**Keywords:** NCD, Transpeople, Transgender and gender diverse, Gender, Inequity, Discrimination, UHC, Risk factors

## Abstract

**Background:**

Non-communicable diseases (NCDs) are high on the priority list of the Kerala government, and exploring the extent to which transgender and gender diverse (TGD) community members benefit from the services of national programmes for NCDs can provide valuable insights on improving the inclusivity of the health system as it moves towards Universal Health Coverage. This study was conducted to explore the prevalence of NCD risk factors as well as facilitators and barriers to NCD management among the TGD population in Kerala.

**Methods:**

A multiple methods study, including a cross-sectional survey of 120 self-identifying TGD people that included an adaptation of the WHO STEPS questionnaire, as well as in-depth interviews with thirteen individuals, was conducted in three districts of Kerala to explore the barriers and facilitators to NCD prevention and management.

**Results:**

The results are presented using the key dimensions emerging out of the Diederichsen framework. A range of discrimination faced by TGD people in Kerala traps them in situations of low educational outcomes with consequent disadvantages in the job market when they search for livelihoods. This results in a large proportion of our sample living away from families (69 percent), and finding themselves in precarious jobs including sex work (only 33 percent had a regular job), with all these aforementioned factors converging to marginalise their social position. This social position leads to differential risk exposures such as increased exposure to modifiable risk factors like alcohol (40 percent were current alcohol users) and tobacco use (40.8 percent currently used tobacco) and ultimately metabolic risk factors too (30 and 18 percent were hypertensive and diabetic respectively). Due to their differential vulnerabilities such as the discrimination that TGD people are subjected to (41.7 percent had faced discrimination at a healthcare centre in the past one year), those with higher exposure to risk factors often find it hard to bring about behavioural modifications and are often not able to access the services they require.

**Conclusions:**

The disadvantaged social position of TGD people and associated structural issues result in exacerbated biological risks, including those for NCDs. Ignoring these social determinants while designing health programmes is likely to lead to sub-optimal outcomes.

## Background

One of the key challenges in designing and implementing Universal health coverage (UHC) is that the capacity of individuals and groups to benefit from health system interventions, depends on more than just the availability and quality of services [1]. A range of social determinants of health have been described that play a key role in the attainment of health [2] and in fact, health systems are considered as an intermediate determinant of health. Health systems can reduce health inequity by addressing the needs and context-specific circumstances of marginalised populations, in order to provide redistributive health care and offer them preferential gains [3]. Since health systems are not purely technical systems but socio-technical systems, the achievement of UHC has to take these factors into account explicitly. We explore these issues through research which focused on the transgender community and their experience with Non-Communicable Diseases (NCDs) in the state of Kerala, which has led the epidemiological transition in India [4, 5].

The word transgender, and the phrase Transgender and gender diverse (TGD) are adjectives which are used to describe a person whose gender identity is incongruent with the sex they were assigned at birth. Trans women are people who are assigned the sex “male” at birth, as well as the associated gender role, but do not identify with these, and identify as women. Trans men are people who are assigned the sex “female” at birth, as well as the associated gender role, but do not identify with these, and identify as men. Gender fluidity is a gender identity that shifts over time and across situations [6].

A wide variety of TGD identities are present in India – including Hijras, Aravanis, Jananas, Kinnars, Kothis, Jogtas/Jogappas, Dhuranis and Shiv-Shakthis. In the British era, same-sex behaviour was a punishable offense (377 of Indian Penal code,1860). However, in 2014, with the National Legal Services (NALSA) judgment, all citizens were given the right to self-identify their gender, and the struggle of TGD people was recognised as a human’s rights issue. Kerala was the first Indian state to execute an exhaustive policy for transgender people as per the decree of the NALSA judgement [7, 8].

There is an abundance of data on structurally contingent elements surrounding health risks of TGD people such as both economic and social marginalisation, pathologization, stigma and ensuing discrimination and even violence faced in different environments including health care centres [9]. The relationship between discrimination and health of TGD people has been explained using a number of theories including the minority stress theory which states that repeated discrimination and transphobic incidents can have deleterious effects on both their physical and mental health. The social exclusion the community faces can lead to social vulnerability, which can have multiple dimensions and operate at both macro and micro levels, often robbing the community members of both their agency and personhood [10, 11]. This web of social inequalities has been shown to result in a number of detrimental health effects, one of which is the high prevalence of risk factors for non—communicable diseases (NCDs) in the TGD community [12].

NCDs are chronic diseases of long duration that cannot be transmitted from one person to another, and are normally caused due to a combination of genetic, physiological, environmental and behavioural factors [13]. The Sustainable Development Goal (SDG) 3.4 aims to attenuate deaths from NCDs by one-third through prevention and early detection by 2030 [14]. This can only be done by exploring the convergence of social, behavioural and demographic factors – from the micro level up to the macro level – with input from key stakeholders in the system [15].

India too has declared its concern with NCD control with the launch of the National Programme for Prevention and Control of Cancer, Diabetes, Cardiovascular diseases and Stroke (NPCDCS) in 2010 [16]. The Indian state of Kerala, with its aspirational health indicators, was one of the first states to detect an advanced epidemiological transition, and efforts to tackle NCDs began in 2009 with the launch of the National Rural Health Mission. Even before this however, local governments who had the power to act as per community requirements due to the decentralised governance system followed in Kerala, have implemented innovative programmes in their localities to control NCDs. With the launch of NPCDCS, India Hypertension Control Initiative (IHCI) and the Aardram mission, Kerala further exhibited its resolve to fight NCDs [17]. Assessing the degree to which the transgender community is benefitting from currently available and highly prioritised NCD services, can provide invaluable and practical insights into making the health system more inclusive, as well as enhancing the effectiveness of these programmes, which will help in the realisation of UHC and the achievement of SDGs.

As per the Kerala State policy for TGD there are an estimated 25,000 TGD individuals in Kerala [8]. TGD people have been shown to be more prone to having NCD risk factors and the use of different forms of tobacco, such as cigarettes and smokeless tobacco has been shown to be higher in TGD people as compared to cis-gender people [18]. Even though there are no previous studies conducted in Kerala on the prevalence of NCD risk factors in the TGD community, literature shows that in other states of India, in addition to having difficulties in accessing health care, they also have a higher prevalence of NCD risk factors [12]. Addictive substances such as tobacco and alcohol are often used as a maladaptive coping mechanism to deal with daily stressors and furthermore, the use of hormone therapy has also been associated with increased risk for venous thromboembolism [19]. The disease burden in the TGD community is magnified because of multiple other factors, such as lack of access to quality health care in a timely manner, lack of empathetic and knowledgeable health care providers and of course the cost of health care in the context of high out-of-pocket expenditure [20]. Studies from Brazil report similar negative findings [21]. TGD people face a multitude of barriers, including individual level barriers such as real or perceived discrimination, interpersonal level barriers such as lack of health care workers who are culturally competent and organisation level barriers such as inappropriate registration forms [20]. By and large, the general healthcare needs of the TGD community can be attended to at the primary health care level, with no difference from those services offered to cis-gender patients [22], and since NCDs are on the rise in Kerala [23], it is vital that these services are accessible to the TGD community so that equitable and effective interventions can be put into place.

Only 0.1 percent of articles in public health research over a period of twenty years in the Medline database involves LGBTQ people [24], and in these articles, mental health is the most studied area followed by sexual health [25]. Research on other key areas of health, such as on NCD risk factors is lacking, especially in Kerala. Research that can enhance the capacity of the health system to cater to the needs of TGD people should be a priority [20] and this research should inform solutions that are acceptable to both the medical and the TGD community, thereby enabling both horizontal and vertical equity [26]. This is especially important since WHO has reported that upto 25% of health inequities are caused by lack of access to effective services [27]. Collaborating with the TGD community to generate high quality data, can contribute to enhancing health and health care access for the community.

## Methods

### Study setting

The state of Kerala, located on India’s tropical Malabar coast, contributes to 38,863 sq. km of India’s land area. Kerala ranks first among the states with regard to the human development index and rural social development index. The “Kerala model of development” has won international acclaim for its sustained performance with regards to basic indicators such as infant mortality rate, literacy and availability of primary health care, which gave rise to an expectation of lower inequity in health and education outcomes. Following the 2014 Supreme court judgment, commonly called the NALSA judgement, that affirmed the legal identity and constitutional rights of TGD people [28], Kerala was the first state to announce a comprehensive transgender policy [29–31]. A sum of INR 10 crore (approximately USD 1.2 million as per the 2023 conversion rate) was set apart for the welfare of the TGD community. In addition to other social welfare schemes, a literacy mission to ensure education with reservations at universities and refunds for sex reassignment surgeries were also initiated, with District and state transgender boards and transgender cells being established to address the needs of the community [32].

The major objective of this study was to determine the proportion of TGD people above the age of 18 in Kerala who had risk factors for NCDs and their determinants. We focused on modifiable risk factors such as tobacco and alcohol use, unhealthy diet, inadequate physical activity, being overweight and obesity and metabolic risk factors such as hypertension and diabetes. The secondary objective was to study the extent to which NCD screening and follow-up services were utilized and accessible to the TGD community. Herein after, the TGD community will be referred to as ‘the community’ in atleast some places in this manuscript, since that is a term often used by TGD people in Kerala to describe their social support networks.This community-based study of TGD people in Kerala used multiple methods including a cross-sectional survey for the quantitative aspect and in-depth interviews for the descriptive qualitative study. The study covered three districts of Kerala, namely Ernakulam, Trivandrum and Kollam, which were purposively selected due to the relatively high number of registered community members. Self-identified TGD people were included and those who did not consent were excluded from the study.

As per information gleaned from conversations with officials at the Kerala Social justice department (SJD), the total number of registered TGD people across Kerala is 1200. Based on the literature review, the proportion of TGD people who use tobacco is 45% [12], which is a recognised risk factor for NCDs, the use of which is well documented in the TGD community [33, 34]. The sample size estimated using a 95 percent confidence interval and a precision of 10 percent in OpenEpi software, was 89. Anticipating non-response and errors the calculated sample size was over sampled by 30%, resulting in a final sample size of 120. For the qualitative aspect, purposive sampling of 13 TGD community members was done based on axes such as gender identity (transwomen/transmen), age (younger/older), comorbidities (present/absent) and experience with discrimination (has experienced/ has not experienced). Occasions where community members gathered were sought out and frequented to invite individuals to join the study. These included medical camps conducted by the National Health Mission (NHM) and self-help group gatherings. This was done by forming and maintaining close working associations with community members employed by the social justice department and community-based organisations, and involving them during the recruitment process. Some respondents who were identified through snowball sampling were visited at their homes, after being introduced through proper channels i.e. mainly through other community members who were mutual friends, the community members willingly and warmly invited the principal investigator into their homes, except in a few instances, some even taking on the responsibility of ensuring that other community members in the vicinity attended these meetings.

Drawing on suggestions that emerged from initial discussions with community members and social advocates regarding their experiences with health care, a structured interview schedule was developed to assess socio-demographic factors, presence of risk factors for NCDs and screening history for NCDs. The structured interview schedule which was developed in English and translated to Malayalam was shared with advocates of the community and based on their recommendations, further changes were made to the tool. NCD risk factor assessment was based on the WHO STEPS questionnaire for NCD surveillance. The Global Physical Activity Questionnaire (GPAQ) was used to assess physical inactivity. Anthropometric and biochemical measurements including height, weight, blood pressure (BP) and blood glucose were measured. (see Appendix 1 for operational definitions). In-depth interview guides were also developed to explore and collect data on health seeking behaviour and barriers; knowledge and practices regarding risk factors; and challenges to lifestyle modification.

The data was analysed using SPSS version 25 software. Univariate analysis was used to analyse the variables. The independent variables were subjected to bivariate analysis using the chi-square test and significant or near significant variables were included for logistic regression analysis. A hybrid coding method was used to analyse the in-depth interviews. The first two interviews, which were selected as they were deemed to be having rich content, were open-coded inductively. The codes created were grouped into axial codes which were further categorized and organized into themes. The remaining interviews were deductively coded and the few portions of the transcripts that could not be included in the pre-existing codes, were incorporated as new codes which contributed to the final themes [35].

## Ethics

The study was undertaken after getting clearance from the Institutional Ethics Committee (IEC) of Sree Chitra Tirunal Institute for Medical sciences and Technology (IEC Regn No. ECR/189/Inst/KL/2013/RR-21) which follows national guidelines as prescribed by Central Drugs Standard Control Organisation (https://cdsco.gov.in/opencms/opencms/en/Home/). The IEC clearance (SCT/IEC/1818/JANUARY/2022) was obtained on 02–03-2022. Permission was also obtained from the State Mission Director of the NHM, in order to attend and collect data at the camps conducted by them. Confidentiality of the collected data was ensured, with any information that could be used to identify participants being deleted at the end of the study. The participants were given a physical copy of the Malayalam version of the consent form and information sheet, which was read aloud to those who couldn't read it. The study's voluntary nature, aims, and possible advantages and risks were explained to participants. Interviews were conducted at mutually agreed upon locations in an environment that the respondents were comfortable in. Starting from the design phase of the study, connections were established with social advocates of the community, both people from within and outside the community, who were vocal about the issues that community members faced and the feedback received was incorporated into the study. This included advocates such as leaders and members of community-based organisations, cis-gender activists of the community and employees at the Transgender cell at the Social Justice Department. Care was taken to use non-stigmatising language in a sensitive manner that accurately reflects the diversity of the community, while also ensuring that the research does not promote conversion or reparative therapy so as to avoid research abuse.

## Analytic framework

To understand and explore potential avenues for intervention in the transgender community, is important to delve on the root of the inequity, which is their marginalised social position.

The central question for Universal Health Care is how to ensure access to quality health care to all members of the society, and in addition, crucially, to ensure that access to that care translates into positive health outcomes including a reduction in socially determined gradients in health outcomes. In the results section, we employ the Diederichsen model, to explain inequities in health in the TGD community that emerged from the findings of this study [36].

The Diederichsen model, which provides a compelling account of the influence of social position on health inequities, presents five mechanisms. Each of these five mechanisms presents an opportunity to favourably impact health inequity through policy changes. The first mechanism is social stratification which can lead to marginalisation of certain groups. Second, these different social positions lead to differential exposures to risk factors and economic and environmental conditions. This in turn leads to the third mechanism, differential vulnerability. Causes of disease act synergistically and a clustering of causes is often seen in the marginalised. The fourth mechanism is differential disease consequences which is affected by ability to access health care and other factors such as availability of stable incomes. Diseases, thus, have different consequences for different individuals and / or groups in society [37].

## Results

The results of the study will be presented using the key dimensions emerging out the Diederichsen framework. In this paper we focus on the aspect of access to health care—and thus show how the differential social position contributes to both differential exposure to risk as well as differential vulnerability to disease due to discrimination and low social position.

### Social context and social position

**Fig. 1:**
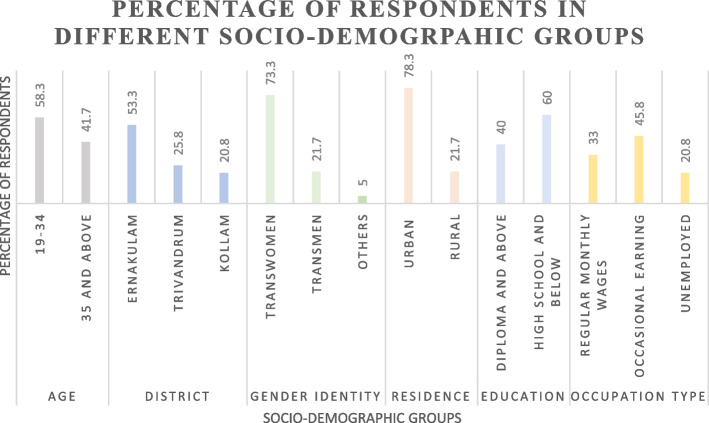
Percentage of respondents in different socio-demographic groups

The baseline characteristics of the study participants are shown in Fig. 1 and Table 1 in Appendix 2.

The mean age of the participants was 33(SD = 8.8) with a 95% CI [32, 35], ranging from 19 to 58 years. 53.3, 25.8 and 20.8 percent of the respondents were from Ernakulam, Trivandrum and Kollam districts respectively. 73.3 percent identified as transwomen, 21.7 percent as transmen and 5 percent as gender fluid. 78.4 percent lived in urban areas and only 30.8 percent were staying with family. More respondents in urban areas (40.4 percent) stayed with friends, than in rural areas (15.4 percent) (Fisher’s two-tailed *p* < 0.001), with majority (65.4 percent) in rural areas living with their own families. Lack of safe spaces for accommodation was a major grievance reported in the qualitative interviews, with respondents reporting they were either denied accommodation or overcharged for it.

Around 48 percent of the respondents currently had life partners. Only 40% had an education of High School level or above. In the qualitative interviews, the reasons attributed to the low levels of education was the discrimination faced by community members at schools and colleges, even at the hands of teachers, while going through the turbulent phase of trying to comprehend their own gender identities. Only 33% reported having a regular job—the rest were employed only occasionally or reported being unemployed and the median income was 10,000 INR (121 USD) with a range from 0–75,000 INR (0–910 USD) and a 95% CI [10,000–15,000]. The qualitative interviews showed that the ubiquitous discrimination that TGD people faced also affected their ability to access livelihoods.

*“We are struggling for 1 Rupee. No one will give us jobs easily. They will think 10 times before giving us a job.”* (IDI-3, transwoman, Trivandrum, 35 years).

### Differential exposures

A high prevalence of modifiable risk factors for NCDs was seen in the sample.

## Prevalence of NCD risk factors

### Behavioural risk factors

49((40.8 percent), 95% CI [32.0,50.2]) currently used tobacco in some form. Only 7.7 percent of those living with their families were daily smokers, while 28 percent of those either living with their friends or in other arrangements were daily smokers. [χ^2^ (2,120) = 6.650, *p* = 0.036]. Exploring these associations with binary logistic regression revealed that compared to those living with their families, those living on their own for rent or in shelter homes had 4.7 times increased odds of being a daily smoker (COR 4.7, 95% CI [1.1, 18.5]. (see Tables 2 and 3 in Appendix 2).

While most of the respondents were generally conscious of the fact that tobacco use had damaging effects on health, only a few seemed to appreciate its specific relation to NCDs, as is illustrated in the below quote:

*“It will slow down hair growth … when we take hormones. When I asked, she (doctor) said like this (sic). She said there will be side effects (to tobacco use). We might get skin problems like pimples … I got acne in between*.” (Field Notes, transman, Trivandrum, 25 years).

The number of current alcohol users was 48(40 percent, 95% CI [31.2, 49.3]). In the qualitative interviews, respondents reported using alcohol as a coping mechanism to deal with stress and also reported being forced into the habit.

*“I started drinking few years back because some clients force us to. They are paying us money … so we can’t say no.”* (IDI-4, transwoman, Ernakulam, sex worker).

As was seen in tobacco use, alcohol use too was seen more in those living with friends (57 percent) than those staying with family (25.6 percent) [χ ^2^ (2, 120) = 8.767, *p* = 0.012)]. As compared to transmen, transwomen had 2.9 times increased odds of using alcohol currently. (COR 2.908, 95% CI [1.066–7.935]) (see Tables 4 and 5 in Appendix 2).

117 (97.5 percent, 95% CI [92.9–99.5]) had less than the recommended quantity of vegetables and fruits per day. Both finances and personal preferences were stated as reasons for this in the qualitative interviews.

*“I eat fruits very rarely … only when I can afford it”* (IDI-3, transwoman, Ernakulam, 35).

The mean number of days that processed food was consumed in a week was 3.4(SD = 2.62), (95%CI [2.93, 3.87]. A one-way analysis of variance showed that the effect of occupation on processed food consumption was significant, [F (2,117) = 3.26, *p* = 0.042]. The mean number of days that processed food was consumed in the unemployed group was more [4.25(SD = 2.574)] than in those with regular wages [3.6(SD = 2.476)].

53(44.2 percent, 95% CI [35.1, 44.2] were found to be physically inactive. Apprehension regarding the lack of finances and the potential discrimination at public places, as well as an unconcerned attitude or restrictions imposed by family in some cases were ascribed as reasons through the qualitative interviews.

“*The gym opens by 5… but at that time it will be full of boys… we won’t get the … freedom to work out…”* (IDI-5, transman, Ernakulam, 26 years).

### BMI and obesity

The mean BMI was 24.24(SD = 4.05), 95% CI [23.5, 24.9]. 32(26.7 percent), 95% CI [19, 35.5] of the total population was found to be overweight. 13(10.8 percent), 95% CI [5.9, 17.8] was found to be obese.

### Prevalence of hypertension and diabetes

33(30 percent), 95% CI [19.7, 36.4] were found to be hypertensive. Out of these, 14(11.6 percent) were unaware of their hypertensive status.

19(15.8 percent), 95% CI [9.8, 23.6] were found to be prediabetic. As compared to those living with their families, those living in shelter homes or own their own had five times increased odds of being prediabetic. (AOR = 5.024, 95% CI [0.971, 25.996]) (see Table 6 in Appendix 2).

22(18.3 percent); 95% CI [11.9, 26.4] were found to be diabetic, out of which 7(5.8 percent) were not aware of their status. As compared to those who were physically active, those who were physically inactive had two times increased odds of being diabetic. (AOR = 2.7, 95% CI [1.0–7.5]). Those who were obese had 5.2 times increased odds of being diabetic as compared to those who were not obese. (AOR 5.2, 95% CI [1.4, 18.1]). (see Table 7 in Appendix 2).

### Differential vulnerabilities

The differential exposures and consequences faced by the community compound the differential vulnerabilities faced by them. The increased vulnerability of this group to the risk factors described above to a significant amount is due to lack of access to health care services and health literacy. These vulnerabilities include having to face discrimination at institutions such as health care facilities, which further negatively affected health care seeking behaviour in the community.

## Screening practices for NCDs

In the one year preceding the study, 90 (75 percent) had their blood pressure checked. Most, [69(92 percent)] of those who had been screened for hypertension in the past one year had done so at a private centre – either as a part of regular follow up of Hormone Replacement Therapy (HRT) or at camps organised by various non-governmental organisations. Out of the total participants, 10(8.3 percent) had not had their blood pressure checked in the five years preceding the study.

In the one year preceding the study, 86 (71.7 percent) had their blood sugar measured. Most, [68(79 percent)] of those screened in past year for diabetes had their blood sugar measured at private centres, while 12(10 percent) had not had their blood sugar checked in the five years preceding the study.

All those who were newly diagnosed with diabetes during the study had reported facing discrimination at a health care centre in the past one year when asked about the same. (Fisher’s two tailed p = 0.041). (see Table 8 in Appendix 2). This association was substantiated in the qualitative interviews, where it was conveyed that a fear of discrimination and internalised stigma could be contributing to avoidance of routine screening.

*“There are many community (members) who just buy some medicines and don’t go to the hospital because of their previous experiences with transphobic doctors. … this is what most people do. (treat themselves at home) … There are still a lot of people who have not changed from the old times, many doctors are there. So, those who have faced them are now hesitant to go to any hospital”* (IDI-6, transwoman, 33 years, Ernakulam).

Even though the large majority of respondents had been screened for NCDs in the one year preceding the study, in the qualitative interviews, the community members generally associated the term screening with sexually transmitted diseases (STDs) and thus, most of the screening for NCDs happened mainly at the initiative of the health system, among those who were on HRT or at camps. This could be due to the fact that for many years, the primary focus of health care services for the community was the control of STDs, which could also cause further marginalisation by stigmatising the TGD community.

*“I don’t know much about that (NCDs)…we are more concerned about syphilis and other skin issues. My BP only increases when I am tensed… otherwise it is normal. Actually, I get tensed when I go to a hospital and see things like syringes… that is when my BP increases (laughs)*” IDI-6, 33 years. transwoman, Ernakulam.

In response to a question on overall discrimination, 50(41.7 percent) reported feeling discriminated against at health care centres in the past one year. (see Table 9 in Appendix 2). More people who were unemployed (52 percent) than those with regular monthly wages (25 percent) reported being discriminated against (χ2 (2,120) = 6.917, *p* = 0.031). More people who were living with their friends (60 percent) than those living with their families (21 percent) reported being discriminated against. (χ^2^(2,120) = 12.750, *p* = 0.002). This could be attributed to the fact that those living with their families often went to centres where they had long standing relations with staff, and they were often also accompanied by their family members.

“There is a clinic in (*) – I have been going there since a young age. They know me since many years so it is comfortable … he is like a family doctor…even all the nurses treat me well.” (IDI-8, transwoman, Ernakulam,38 years) (*) – village name.

More people who had an income below 10,000 INR had experienced discrimination than those with an income above 10,000 INR. (χ^2^(1,115) = 6.069, *p* = 0.014).

More people in the higher income group used private centres as compared to the lower income group (40.9 vs 30.6) even though there was no statistically significant association between centre used and income. (χ2 (2,115) = 2.469, *p* = 0.291).

Most respondents, [66(55 percent)] felt there was a paucity of health care providers who were friendly to TGD people. Discrimination at healthcare centres was experienced in many forms by the community, including rushed consultations, being refused treatment and delay in receiving treatment.

*“Earlier (before transition), the doctors used to place the stethoscope on our chest and see if we have any breathing difficulties … they used to understand what our illness is. Now, they will just ask if we have any allergy for any medicine. As soon as we say no, they will write a lot of medicines and give us.”* (IDI-9, transwoman, TRV, 35 years).

Receiving delayed treatment was a frequent grievance and some respondents reported being asked to wait till all the cis-gender people were attended to before they received treatment. Reports of having faced refusals to provide treatment surfaced too, especially in services highly demanded by the community such as HRT and sex reassignment surgeries (SRS), but also in one case during an attempt to screen for diabetes. Difficulties caused due to other health care seekers were commonly reported across interviews. The demeaning looks and comments that cis-gender people subjected the TGD population to was a source of great anxiety for many respondents, though some reported having developed resilience to it.

“*We hesitate to go to government hospitals because of this … the way people look at us like we are some animals that came out of somewhere … because we feel bad most of us don’t go to government hospitals*.” (IDI-8, transwoman, Ernakulam, 38 years).

### Differential consequences:

Many of those who had undergone NCD screening reported that they only did so when they experienced symptoms. Even once diagnosed with a NCD, many of the community members did not regularly attend follow up appointments as illustrated by the quote below by a respondent who was previously diagnosed with hypertension.

*“I didn’t feel anything. I didn’t feel any problems with my body. I didn’t get a fever or anything… so I didn’t do it(follow-up).*” (IDI-8, transwoman, Ernakulam, 38 years).

Financial issues and difficulty in finding a TGD friendly hospital were also cited as reasons for not undergoing screening and follow-up for NCDs. Thus, there was a lack of continuity in care, including a lack of follow-up for complications which could potentially lead to worse consequences.

**Fig. 2:**
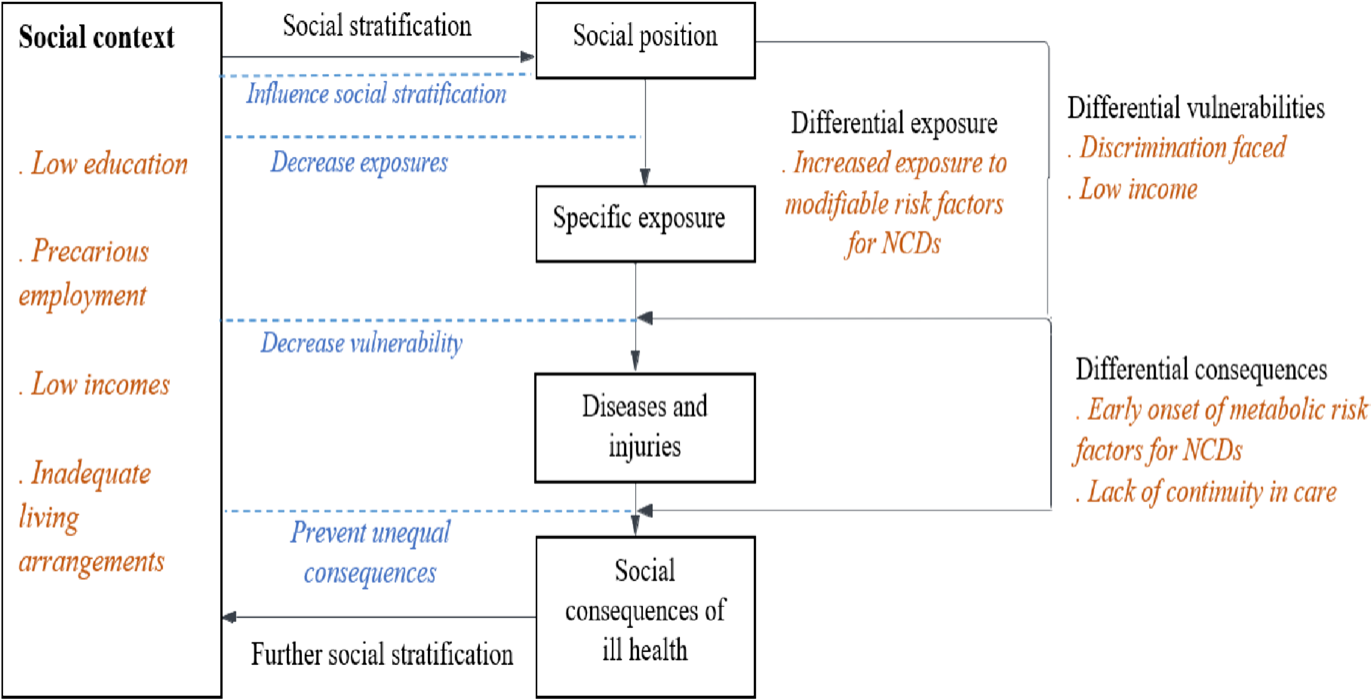
Results and broad areas of recommendations (in italics) of the study represented in the Diederichsen model

To summarise, as shown in Fig. 2, the marginalised social position of the TGD community has its genesis in the prevalent heteronormative social context in Kerala, which often resulted in a lack of family support, low education and employment opportunities and inadequate living arrangements for the community members. This relegated them to a lower social position which in turn led to differential exposures and consequences such as a high prevalence of modifiable risk factors for NCDs and an early onset of metabolic risk factors. Ultimately TGD people are exposed to vulnerabilities such as having to face discrimination at institutions including health care facilities, which further negatively affects their health.

## Discussion

**Fig. 3:**
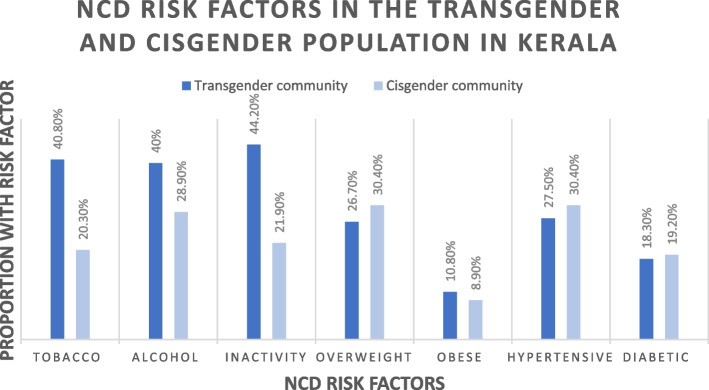
Proportions of NCD risk factors in the TGD and cisgender population [39] in Kerala

Overall, the results show a higher prevalence of risk factors among the TGD community that was surveyed as compared to the cis-gender population in Kerala (Fig. 3), and this adverse risk profile of the TGD community is occurring in the context of a marginalised social position as detailed in the baseline characteristics The fact that 60 percent had an education at high school level or below is of significance since Kerala is renowned for having the highest literacy rates, along with being better positioned with relation to school drop-out rates in India [38].

With regards to tobacco and alcohol use respectively, 40.8 and 40 percent of the study sample were current users, which is similar to, and in some cases lower than, the findings in previous studies done in the TGD community in other states of India [12, 18, 40–42]. In the cisgender population in Kerala, the prevalence of tobacco and alcohol use is lower, at 20.3 and 28.9 percent respectively [39]. Both tobacco and alcohol use were more in those living away from family, thus implicating the importance of social support in mitigating harmful substance use.

In our sample, 97.5 percent possessed an unhealthy diet which again, is similar to previous studies [12], with processed food consumption being more in the unemployed group. Even though the levels of physical inactivity in our study were lower than those in previous studies in the TGD community, it was much higher than the cis-gender population in Kerala (21.9 percent) [43].

While the prevalence of being overweight was similar or more, and the prevalence of obesity was either similar or lower than previous studies in India [12], it was comparable to those of the cisgender population in Kerala [39].

The prevalence of hypertension and diabetes detected in the study was similar to that of the prevalence in the cisgender population in Kerala. However, it is crucial to note that the mean age of the respondents in the cisgender study [42.5(SD = 14.8)] was around 10 years more than that in our study [33(SD = 8.8)], thus suggesting an early onset of NCDs in the TGD population.

In this study, the quantitative aspect reveals that quite a large proportion of the TGD community had their blood pressure and blood sugar levels checked in the last year, with 75 percent and 71 percent having undergone check-ups for hypertension and diabetes respectively in the past one year, which is comparable to previous studies [12]. However, as revealed with the qualitative study, most of this screening happens in camps or among those who are on HRT at the behest of the health system, with 11.6 and 5 percent being unaware of their hypertensive and diabetic status respectively. Importantly, the community seemed more aware about STD’s and related screening than NCDs, probably as this was the sole focus of health services towards the community in the last few decades. Certain features of the health system revealed concerning trends from the perspective of the evolution of a comprehensive patient centred screening program as envisaged in the NPCDCS. Thus, a significantly large proportion of individuals reported discrimination and individuals recounted numerous instances of poor-quality care reflecting a discriminatory and insensitive attitude towards members of the community. A large proportion (41.7) percent reported feeling discriminated against at a health care centre in the past year. Even though this is a decline from the 51 percent who reported having faced discrimination at health care centres in the SJD survey in 2015, it is still disheartening [8]. Thus, while some minimal services such as screening are provided in isolated situations, for long-term care, the health system is still discriminatory. In the case of chronic conditions like NCDs, life-long surveillance is required, mandating that regular follow-ups be done to assess modification in diet, exercise and other risk factors. Unfortunately, even though many community members report having their blood pressure checked at the behest of the health system, adequate awareness about its significance and regular follow-up in the community is affected by discrimination and lack of knowledge. Thus, the poor social position which results in a higher risk profile, is exacerbated by lack of access to health care facilities. The health system, which would ideally be expected to overcome this burden, however seems to be failing to do so.

At the outset, a range of discrimination faced by the TGD community in Kerala – including from their families and as they struggle with their gender identities during their school years, traps them in situations of low educational outcomes with consequent disadvantages in the job market when they search for livelihoods. This results in a large proportion of our sample living away from families, and finding themselves in precarious jobs including sex work, with all these aforementioned factors converging to marginalise their social position. It is critical to appreciate the way in which this societal discrimination causes the TGD community to slide down, and become trapped in the risky stigma-sickness slope [44]. This social position leads to differential risk exposures as seen in our sample, with increased exposure to modifiable risk factors such as alcohol and tobacco use and ultimately metabolic risk factors too. Due to their differential vulnerabilities such as the discrimination that community members are subjected to, those with higher exposure to risk factors often find it hard to bring about behavioural modifications and those having a higher need for health system inputs are often not able to access the services they require. In addition to discrimination, financial issues arising out of their marginalised social positions, also play a role in increasing the vulnerability of the community since lack of finances was described as a major barrier to affording a healthy lifestyle, accessing timely health care and a major determinant in choosing a centre as well. The health system barriers ensured that those susceptible to risk factors and those with diseases could not access necessary services. These differential vulnerabilities further lead to a higher rate of conversion of these risk factors into disease due to the barriers to access quality health care services described above, as has been seen with the early onset of metabolic risk factors like hypertension and diabetes in our sample. A crucial finding unearthed in our study was the apparent beneficial consequences of having family and social support, as risk factors like tobacco and alcohol use and chances of having reported discrimination was more prevalent in those living away from their families. Similar findings have been seen in studies in the United States of America [45].

While UHC approaches tend to focus on health care provision and financial protection, very few engage with the larger determinants of health which prevent marginalized groups from accessing and benefitting from these interventions. Thus, despite having one of the best public health systems in the country, and boasting remarkable average health indicators, the state’s public health care system is perceived as a space of discrimination by many members of the TGD community. Despite, achievements in education and policies for social inclusion, our study reveals the marginalization that the TGD community continues to face. The continued gaps highlight the limitations of a purely technical approach to UHC, and underlines that “leaving no one behind” requires a much more inclusive, comprehensive and inter-sectoral approach.

Strengths and limitations: To the best of our knowledge, this is the first study in Kerala, done to assess risk factor prevalence in the TGD community and barriers to effective screening and follow-up of NCDs. The participants were drawn from three different districts in Kerala thus enabling reasonable geographic representation. Data collection was done only by the principal investigator, thus eliminating inter-observer bias. Most questions asked were regarding the past one year such as tobacco use, screening and discrimination, thus avoiding recall bias. The principal investigator endeavoured to avoid offensive, stereotypical or leading language and this was assisted by feedback from the community advocates during the design phase of the study. Standard calibrated instruments were used for all respondents, thus eliminating the possibility of systematic technical bias. Most importantly, the mixed methods approach adopted in this study enabled a deeper understanding of the results, with both methods supplementing each other. The study had a few limitations as well, primary being the sampling technique. Given that the TGD community is a stigmatised and marginalised community, we had to rely on self-selection and snow-balling to recruit the participants, which would lead to some inevitable biases. Our sample included only those consenting individuals attending camps, and thus, there is a likelihood that those who are in the lowest ranks of the community have been underrepresented. There is also a possibility of social desirability bias, with underreporting of modifiable risk factors being a possibility.

## Conclusion

In general, a larger proportion of the TGD community members in our sample had modifiable risk factors as compared to the cis-gender population in Kerala. Additionally, there was an apparent early onset of metabolic risk factors like hypertension and diabetes. A major proportion of the respondents had undergone screening for diabetes and hypertension in the past one year. Given insights from the Commission on the Social Determinants of Health and the findings reported here, it is clear that larger structural issues are exacerbating biological risks. In such a situation, neglecting these social determinants in the design of health programmes, is likely to lead to sub-optimal outcomes. The key take-away for us is the limitations of seeing the path towards UHC as one that can be traversed once we have the right mix of technologies and insurance. Our study clearly shows that the ability to benefit from these interventions is itself determined by a complex set of social and societal determinants.

The results emerging from this study force us to desist from making purely technical or health system focused recommendations. It reiterates the importance of engaging with the determinants of social position and stigma – which are outside the scope of health interventions, but are things that the health system should advocate for – especially in the drive toward UHC.

The recommendations arising from this study can also be explained using the Diedericshen model. Specific attempts for improving health literacy on the various risk factors and other conditions affecting the TG community should be undertaken in partnership with the community. Since many members reported undergoing frequent check-ups for the detection of STD’s, these venues can be used as a convenient location for opportunistic screening for NCDs. In other words, there is a need for the development of comprehensive services preferably merged with the general health services, or sensitively designed to meet the specific needs of the local communities. Creating safe spaces including peer support groups in order to provide aid in tobacco and alcohol cessation can be attempted. When attempting to decrease the vulnerability of the community, it is important to judiciously identify multiple marginalities within the group and design interventions for the more vulnerable subsets of the population. Since there seems to be an apparent early onset of NCDs in the community, there is a need to initiate screening programmes which include those below thirty years of age.

Medical education at the graduate level should include significant components that would give newly minted doctors the confidence to treat TGD people. Additionally, in-service training should be given to health care workers currently in government service and every government hospital should have at least one trained doctor as a contact person, who would bear the responsibility of ensuring appropriate care of every TGD person visiting that centre. In order to prevent unequal consequences, the specific requests and needs of the community should be catered to in government hospitals, ensuring affordability and ease of access.

In addition to these health system specific recommendations – the research points to the need for larger societal interventions along the same lines. At a more general level, ensuring equal employment opportunities to the community within health care centres, in both technical posts and non-technical posts. This would increase the visibility of the community, thus helping them gain acceptance within the society, as well as encourage other TGD community members to visit these centres. Finally, researchers in public health should strive to become advocates for the TGD community to enable larger and more representative studies, while ensuring that ethical considerations are met. It is pivotal to indulge in participatory research by collaborating with community stakeholders, in order to allow the marginalised voices to emerge.

## Data Availability

The corresponding author will provide the transcripts, data set and analysis of the current work on reasonable request.

## Appendix 1

### Operational Definitions

*Current smoker:* a person was defined as a current tobacco user if they used any form of tobacco within the past thirty days. A person was classified as a daily smoker if they used tobacco products daily in the past thirty days. Smoking tobacco products include manufactured cigarettes, beedi, and pipes full of tobacco, hookah, and cigars. Smokeless tobacco was any product which contains tobacco as a constituent including snuff, chewing tobacco, betel, ghutka, khaini etc.

*Current alcohol use*: Current alcohol use was defined as having atleast one standard drink of alcohol in the past 30 days. A standard alcoholic drink of Regular beer is 285ml, single measure of spirit is 30ml, one medium glass of wine is 120ml and one measure of aperitif is 60ml.

*Inadequate fruits and vegetable intake*: inadequate vegetable consumption was defined as less than five servings of fruit and vegetables per day excluding starchy vegetables. One standard serving of vegetable is 80 grams depending on the type of vegetable and standard cup measure. One medium sized fruit is one serving.

*Physical inactivity*: physical inactivity was measured using the GPAQ and was defined as less than 600 metabolic equivalents of moderate and vigorous intensity activity in a typical week.

*Obesity and overweight*: obesity was defined as having a body mass index of 30kg/m^2^ or greater. A person was classified as being overweight if their body mass index was in the range of 25–29.99kg/m^2^.

*Hypertension*: Hypertension was defined as systolic BP ≥140mm Hg or diastolic BP ≥90mm Hg, or if the person is currently using antihypertensive medication (self-reported treatment). [Indian Guidelines on Hypertension IV (IGH IV)]

*Pre-diabetes and diabetes*: A person was described as pre-diabetic if they had a fasting blood glucose (FBG) in the range of 100–125mg/dL. A person was described as diabetic if their FBG was greater than or equal to126mg/dL, random blood sugar was greater than 200mg/dl or they were on self-reported treatment for diabetes. (American Diabetes Association).

## Appendix 2

### Tables of quantitative data analysis

**Table 1:** Baseline characteristics of the study participants:

		Number	Percentage
Age	19–34	70	58.3
	35 and above	50	41.7
District	Ernakulam	64	53.3
	Trivandrum	31	25.8
	Kollam	25	20.8
Gender identity	Transwomen	88	73.3
	Transmen	26	21.7
	Others	6	5
Residence	Urban	94	78.3
	Rural	26	21.7
Living arrangements	With family	39	32.5
	With friends	42	35
	Others(rent/shelters)	39	32.5
Life partner	Yes	58	48.3
	No	62	51.7
Education	Diploma and above	48	40
	High school and below	72	60
Occupation type	Regular monthly wages	40	33
	Occasional earning	55	45.8
	Unemployed	25	20.8
Income (*N* = 115)	Below 10,000	49	42.6
	10,000 and above	66	55
	Missing	5	4.2

**Table 2:** Association between daily smoking and living arrangements:

Smoking related variable	Background characteristics	*N *= 120	*n* (%) in categories of independent variable	Chi square result
Daily smoking	**Living arrangements**			χ^2^(2,120) = 6.650, *p* = 0.036
	with family	39	3(7.7)	
	With friends	42	12(28.6)	
	Others	39	11(28.2)	

**Table 3:** Factors associated with daily smoking:

Variable	Unadjusted OR* (95% CI)	*P* value
**Living arrangements**		
With family	Reference	
With friends	4.8(1.2–18.6)	**0.023**
Others	4.7(1.1–18.5)	**0.026**

*OR – Odds Ratio

**Table 4:** Factors associated with alcohol use

Background characteristics	*N* = 120	*n* (%) in categories of independent variable	Result of cross tabulation
**Living arrangements**			
Family	39	10(25.6)	χ^2^(2,120) = 8.767, *p* = 0.012
Friends	42	24(57.1)	
Others	39	14(35.9)	
**Gender identity**			
Transman	26	6(23.1)	Fisher’s test (two-tailed *p* = 0.049)
Transwoman	88	41(46.6)	
Others	6	1(33.3)	

**Table 5:** Factors associated with current alcohol use

Variable	Unadjusted OR (95% CI)	*P *value
**Gender**		
Transman	Reference	
Transwoman	2.9(1.0–7.9)	0.037
Others	0.6(.06–6.8)	0.733

**Table 6:** Factors associated with pre-diabetics:

Background characteristic (*N*)	*n*(%)	Cross tabulation result	Unadjusted OR (95%CI)	*P *value	Adjusted OR (95%CI)	*P *value
**Living arrangements**						
Family (39)	2(5.1)	χ^2^(2,120) = 5.215, p = .074	Reference		Reference	
Friends (42)	8(19)		4.3(0.8–21.9)	.075	3.6(0.7–19.2)	0.122
Others (39)	9(23.1)		5.5(1.1–27.6)	.036	5(0.9–25.9)	0.054

**Table 7:** Factors associated with being diabetic:

Independent variable	Unadjusted odds ratio (95% CI)	*P* value	Adjusted odds ratio (95% CI)	*P* value
**Physical inactivity**				
No	Reference		Reference	
Yes	2.6(1.0–6.9)	0.046	2.7(1.0–7.5)	0.047
**Obesity**				
No	Reference	0.010	Reference	
Yes	4.8(11.4–16.3)		5.2(1.4–18.1)	0.012

**Table 8:** Factors associated with being newly diagnosed with diabetes during the current study:

Newly diagnosed diabetics	Discrimination faced	No discrimination	*P *value
Yes	7(100)	0	0.041*
No	63(55.8)	50(44.2)	

**Table 9:** Factors associated with discrimination:

**Background characteristics**	**Total (*****N*****)**	***n*** **(%)**	
**District**			χ^2^(2,120) = 7.253,*p* = 0.027
Ernakulam	64	33(51.6)	
Trivandrum	31	7(40)	
Kollam	25	10(22.6)	
**Living arrangements**			χ^2^(2,120) = 12.750,*p* = 0.002
Family	39	8(20.5)	
Friends	42	25(59.5)	
Others	39	17(43.6)	
**Income**			χ^2^(1,115) = 6.069,*p* = 0.014
Below 10,000	49	26(53.1)	
10,000 and above	66	20(31.3)	
**Occupation**			χ^2^(2,120) = 6.917,*p* = 0.031
Regular wages	40	10(25)	
Occasional earnings	55	28(50.9)	
Unemployed	25	12(52)	
